# Supplementary material for: Asymptomatic and mildly symptomatic influenza virus infections by season -- Case-ascertained household transmission studies, United States, 2017–2023
Source: medRxiv. 2024 Jul 18:2024.07.17.24310569. Preprint. [Version 1] doi: 10.1101/2024.07.17.24310569 (PMC11275689; doi:10.1101/2024.07.17.24310569)
Supplement: Supplement 1 [file NIHPP2024.07.17.24310569v1-supplement-1.pdf]

medRxiv preprint doi: https://doi.org/10.1101/2024.07.17.24310569; this version posted July 18, 2024. The copyright holder for this preprint (which was not certified by peer review) is the author/funder, who has granted medRxiv a license to display the preprint in perpetuity. This article is a US Government work. It is not subject to copyright under 17 USC 105 and is also made available for use under a CC0 license.

Supplemental Table 1. Characteristics of influenza positive contacts by season, United States, 2017-2023

Characteristic	**Overall**, N = 426[1]	**2017-2018**, N = 45[1]	**2018-2019**, N = 107[1]	**2019-2020**, N = 152[1]	**2021-2022**, N = 45[1]	**2022-2023**, N = 77[1]
State						
TN	231 (54%)	24 (53%)	47 (44%)	46 (30%)	45 (100%)	69 (90%)
WI	195 (46%)	21 (47%)	60 (56%)	106 (70%)	0 (0%)	8 (10%)
Sex						
Female	214 (50%)	26 (58%)	52 (49%)	69 (45%)	29 (64%)	38 (49%)
Male	212 (50%)	19 (42%)	55 (51%)	83 (55%)	16 (36%)	39 (51%)
Race, Ethnicity						
Asian, Non-Hispanic	11 (2.6%)	0 (0%)	3 (2.8%)	8 (5.3%)	0 (0%)	0 (0%)
Black, Non-Hispanic	20 (4.7%)	2 (4.4%)	4 (3.7%)	6 (3.9%)	7 (16%)	1 (1.3%)
Hispanic/Latino	69 (16%)	6 (13%)	12 (11%)	13 (8.6%)	16 (36%)	22 (29%)
Multiple race, Non-Hispanic	9 (2.1%)	0 (0%)	1 (0.9%)	6 (3.9%)	2 (4.4%)	0 (0%)
NH/OPI, Non-Hispanic	1 (0.2%)	0 (0%)	0 (0%)	0 (0%)	0 (0%)	1 (1.3%)
Unknown/Refused	3 (0.7%)	0 (0%)	0 (0%)	0 (0%)	0 (0%)	3 (3.9%)
White, Non-Hispanic	313 (73%)	37 (82%)	87 (81%)	119 (78%)	20 (44%)	50 (65%)
Vaccination Status						
Unvaccinated	257 (60%)	26 (58%)	50 (47%)	100 (66%)	25 (56%)	56 (73%)
Vaccinated	169 (40%)	19 (42%)	57 (53%)	52 (34%)	20 (44%)	21 (27%)
Age Group						
0-17	208 (49%)	16 (36%)	60 (56%)	90 (59%)	15 (33%)	27 (35%)
18-49	161 (38%)	19 (42%)	33 (31%)	50 (33%)	18 (40%)	41 (53%)
50+	57 (13%)	10 (22%)	14 (13%)	12 (7.9%)	12 (27%)	9 (12%)
Flu Type						
A, H1	152 (36%)	8 (18%)	41 (38%)	87 (57%)	2 (4.4%)	14 (18%)
A, H3	139 (33%)	24 (53%)	61 (57%)	0 (0%)	29 (64%)	25 (32%)
A, no subtyping	44 (10%)	1 (2.2%)	3 (2.8%)	1 (0.7%)	14 (31%)	25 (32%)
B, no subtyping	82 (19%)	11 (24%)	1 (0.9%)	62 (41%)	0 (0%)	8 (10%)
Unknown	9 (2.1%)	1 (2.2%)	1 (0.9%)	2 (1.3%)	0 (0%)	5 (6.5%)

[1]n (%)

medRxiv preprint doi: https://doi.org/10.1101/2024.07.17.24310569; this version posted July 18, 2024. The copyright holder for this preprint (which was not certified by peer review) is the author/funder, who has granted medRxiv a license to display the preprint in perpetuity. This article is a US Government work. It is not subject to copyright under 17 USC 105 and is also made available for use under a CC0 license.

Supplemental Table 2. Characteristics of the cohort & frequencies of asymptomatic infections with follow-up restricted to 5 days[+], United States, 2017-2023

	Influenza positive contacts	Asymptomatic influenza positive contacts	Modeled Asymptomatic infections*	Contacts without ARI	Modeled Infections without ARI*	Contacts without ILI	Modeled Infections without ILI*
	**N**	**N**	**% (CI)**	**N**	**% (CI)**	**N**	**% (CI)**
**Age Group**							
0 – 17 years	204	11	7 (3-12)	24	15 (9-22)	51	29 (22-38)
18 – 49 years	159	19	12 (7-20)	27	17 (11-26)	79	53 (44-62)
50+ years	57	4	5 (2-15)	10	13 (7-23)	28	48 (35-61)
**Season**							
2017 – 2018	42	3	6 (2-18)	5	11 (5-25)	15	35 (21-52)
2018 – 2019	106	5	4 (2-9)	11	9 (4-17)	34	34 (25-45)
2019 – 2020	150	5	3 (1-9)	8	5 (2-12)	43	34 (25-43)
2021 – 2022	45	7	15 (7-30)	16	36 (22-53)	26	59 (40-75)
2022 – 2023	77	14	17 (9-31)	21	30 (18-46)	40	55 (41-67)
**Influenza Vaccination Status**							
Vaccinated	167	18	11 (7-17)	36	24 (17-33)	75	51 (41-60)
Unvaccinated	253	16	5 (3-9)	25	9 (6-14)	83	36 (29-44)

*A robust logistic regression model accounting for household clustering was conducted to estimate the proportion of influenza positive household contacts that were asymptomatic, without ARI, and without ILI. Adjusted marginal estimates and 95% confidence intervals are shown as percentages. Age group was adjusted for season and vaccination status. Season was adjusted for age group and vaccination status. Vaccination status was adjusted for age group and season.

[+]Follow-up was restricted to 5 days of follow-up which was the minimum collected per site protocol as sensitivity analyses.
